# Joint effects of elevated copper and temperature in juvenile Tambaqui exposed in black and white waters of the Amazon

**DOI:** 10.1111/jfb.70238

**Published:** 2025-10-17

**Authors:** Anne Crémazy, Carolyn Morris, Susana Braz‐Mota, Chris M. Wood, Rafael M. Duarte, Ora E. Johannsson, Adalberto L. Val

**Affiliations:** ^1^ Centre Eau Terre Environnement Institut National de la Recherche Scientifique Québec City Québec Canada; ^2^ Department of Zoology University of British Columbia Vancouver British Columbia Canada; ^3^ Laboratory of Ecophysiology and Molecular Evolution Brazilian National Institute for Research of the Amazon (INPA) Manaus Brazil; ^4^ Biosciences Institute São Paulo State University – UNESP, Coastal Campus São Vicente Brazil

**Keywords:** *Colossoma macropomum*, CT_max_, cumulative effects, ion regulation, Rio Negro, Rio Solimões

## Abstract

This study aimed to investigate how exposure to elevated water temperature and metal concentration jointly affect the physiology of Amazonian fish. Aboard a research vessel in the Amazon, we evaluated the effects of water temperature (river T°C at 31.5°C and a + 4°C increase to 35.5°C) and of 3‐h copper (Cu) exposure (up to 600 μg/L) in juvenile Tambaqui (*Colossoma macropomum*) exposed in freshly collected Rio Negro (‘black water’) and Rio Solimões (‘white water’) waters. In Cu‐free water, the +4°C raise accelerated physiological Na^+^ influx and efflux rates, but only in Rio Negro water. Temperature had no effects on the other physiological fluxes (Cl^−^, K^+^ and ammonia fluxes). Cu exposure led to net losses of Na^+^ (via increased efflux), Cl^−^ and K^+^ and decrease in acute upper thermal tolerance (CT_max_). These Cu effects were more prominent in Rio Negro water, where Cu bioavailability was the greatest. The +4°C change had no effect on gill Cu accumulation and, overall, there was limited evidence that warming worsened Cu‐induced ionoregulatory disturbances. However, in Rio Negro, as Cu and heat both separately promoted Na^+^ net losses, fish Na^+^ balance was the most compromised in the presence of the two stressors. Altogether, the impaired thermotolerance and ionoregulation under combined Cu and heat exposures suggest a cumulative physiological interaction between two stressors that are increasing threats to the Amazon basin.

## 1 INTRODUCTION

Amazonia hosts 15% (>2700 species) of all the freshwater fish species of the world, many of them endemic to the region (Jézéquel et al., 2020). This invaluable diversity is currently under serious threat from multiple anthropogenic pressures, including climate change and pollution (Val & Wood, 2022). Notably, the IPCC (2014) has predicted that baseline water temperatures (currently 28–31°C) could increase by 2.2–7.0°C in Amazonia in the worst‐case scenario of climate change. Further, short‐term heatwave events that greatly elevate water temperatures are predicted to increase in frequency and intensity in this region (Geirinhas et al., 2018). In fact, in 2023, the Amazon experienced an unprecedented drought‐heatwave event, driven by a strong El Niño phenomenon, global warming and deforestation (Espinoza et al., 2024). The ensuing combination of lower dissolved oxygen and high water temperatures (up to 40°C in some areas) led to mass mortality of fish and other aquatic wildlife (Braz‐Mota & Val, 2024). Amazonian fish are particularly at risk from increasing temperature, as recent studies have shown that their normal ambient temperatures (28–31°C) are already close to their upper thermal limits (36–42°C) (Campos et al., 2019; Jung et al., 2020; Lapointe et al., 2018).

Pollution poses another significant challenge to Amazonian fish (Braz‐Mota et al., 2024). Intensifying mining, industrial and agricultural activities and urban expansion are increasing the release of pollutants, such as trace metals, into Amazon water bodies. The expansion of mining in this region is partly due to the increasing global demand for critical minerals essential for energy transition [e.g., copper (Cu) and nickel] (Bispo, 2024; Romani & Samora, 2025). This is particularly evident in the Carajás area in the southeastern part of the Amazon (Para state, Brazil), which holds some of the largest iron, Cu and nickel ore deposits in the world and has large active open‐pit mines extracting theses minerals. In parallel to industrial mining, small‐scale artisanal mining, including illegal mining of Cu, is also increasing in the Amazon (Tollefson, 2021). Water, sediment and biota samples collected in areas close to mines and urban centres showed elevated concentrations of various metals (e.g., Cu, nickel, zinc, lead, cadmium) above CONAMA national guidelines for Brazil, as reviewed by Braz‐Mota et al. (2024), Echevarría et al. (2024) and Moulatlet et al. (2023). Among only six metals studied (cadmium, Cu, lead, manganese, mercury, nickel), Cu was found to have the greatest toxicity to Amazon fish. Exposure to this metal has been associated with disruptions of ion regulation (e.g., net Na^+^ loss), metabolism and oxidant/antioxidant balance in Amazonian fish (Braz‐Mota et al., 2018; Crémazy et al., 2022; Matsuo et al., 2005). The magnitude of these effects is known to be greatly influenced by in situ variations in Cu bioavailability, driven by variations in local water quality [e.g., pH, dissolved organic carbon (DOC) and total dissolved solids (TSS) concentrations] within the Amazonia watershed (Crémazy et al., 2016, 2019; Duarte et al., 2024). Therefore, studies on metal toxicity to aquatic organisms must account for the modifying effects of local water quality. Compared to water chemistry, temperature has been scarcely investigated as a metal toxicity‐modifying factor on Amazon fish (Braz‐Mota et al., 2016).

These increasing temperatures and metal pollution trends in Amazonia highlight the need to evaluate the combined effects of these disturbances on local wildlife. In general, increased temperatures have been found to increase metal toxicity in aquatic ectotherms, whose body temperatures match environmental temperatures (Heugens et al., 2001; Sokolova & Lannig, 2008). A proposed reason for this increased toxicity is via increased metal uptake rates at ion transporter proteins in the epithelial membrane, following the Arrhenius law describing the temperature dependence of reaction rates (Schulte et al., 2011; Sokolova & Lannig, 2008). Another explanation is via increased organism sensitivity under thermal stress (Sokolova & Lannig, 2008). In contrast, elevated metal exposures have been associated with reduced upper thermal tolerance in aquatic ectotherms (Negri & Hoogenboom, 2011; Sokolova & Lannig, 2008). This reduced thermal tolerance might be due to detrimental effects of metals on the aerobic metabolic capacities (e.g., due to energy costs for metal detoxification) (Couture & Kumar, 2003). However, the interaction of metal with temperature is complex, and opposite observations have been made (Kumar et al., 2023; Mottola et al., 2022), possibly due to cross‐protection stressor interactions, whereby exposure to a priming stressor improves resilience to a different stressor (Rodgers & Gomez Isaza, 2021). Overall, more experimental studies are needed to elucidate the joint effects of elevated temperature and metal exposure. Braz‐Mota et al. (2016) showed that the survival of the native Amazon fish *Hoplosternum littorale* was greatly reduced by a 96‐h exposure to 50 μg/L of Cu (in Rio Negro water) at 34°C but not at 28°C. However, the reasons for this increased Cu toxicity could not be ascertained, as Cu bioaccumulation and its main acute toxicity target (Na^+^ regulation) were not measured in this study. Our goal was to advance our understanding on the joint effects of elevated temperature and metal exposure by characterizing the interactions of elevated Cu and temperatures on a model Amazonian fish.

In a study aboard a research vessel in November–December 2023, we investigated the interactive effects of elevated water Cu concentrations and temperature in juvenile Tambaqui (*Colossoma macropomum*, G. Cuvier 1818). This neotropical characiform is an important local food source and is prevalent in regional fisheries and aquaculture. Further, this fish is present throughout Amazonia and migrates annually for dispersion and growth and for reproduction between the two major waters we visited during our research trip: the ‘black waters’ of the Rio Negro [low pH, ion and levels of total suspended solids (TSS) and high levels of dissolved organic carbon (DOC)] and the ‘white waters’ of the Rio Solimões (neutral pH, higher ion and TSS levels and moderate DOC levels) (Val & de Almeida‐Val, 1995). Because of distinct water chemical compositions, we expected different Cu bioavailability and fish ion regulation in these two habitats. Therefore, we conducted our study in these two river waters. We collected water from the Rio Negro and Rio Solimões, away from conspicuous metal pollution sources, and spiked the water with Cu [0 (control), 200 and 600 μg/L). Firstly, we characterized Cu bioaccumulation in gills and effects on ion regulation (Na^+^, Cl^−^, K^+^ and total ammonia) over a 3‐h exposure of Tambaqui to these test waters at ambient water temperature (31.5°C). We hypothesized that the high Cu‐DOC binding in Rio Negro would result in a lower Cu bioavailability and less‐severe physiological effects than in Rio Solimões water, as observed in a previous study on cardinal tetra *Paracheirodon axelrodi* (Crémazy et al., 2016, 2019). Secondly, we evaluated how an acute +4°C increase in water temperature (31.5°C vs. 35.5°C) affected ion regulation and ammonia excretion for the same Cu treatments. We anticipated that this warming would result in higher baseline Na^+^ influx and efflux rates and higher ammonia excretion due to temperatures effects on biochemical rates (Arrhenius law). We also hypothesized that this warming would lead to increased Cu gill bioaccumulation and thus to increased Cu effects. Thirdly, we evaluated how the various Cu treatments affected the upper critical thermal maximum (CT_max_) of Tambaqui. Based on the available literature, we anticipated a reduction in thermal tolerance following Cu exposure.

## 2 MATERIALS AND METHODS

### 2.1 Experimental locations

The study was conducted aboard a research vessel (the *Anna Clara*, Manaus, AM, Brazil) during a November–December 2023 field trip in two locations of the Amazon River basin, away from conspicuous sources of metal pollution (i.e., with relatively low background Cu levels). The first location was the Anavilhanas archipelago of the Rio Negro (~110 km upstream of Manaus; 02°43′10.3′′ S, 060°45′19.6′′ W), which was visited between 22 and 29 November 2023. The second location was close to the municipality of Manaquiri (AM) on the Rio Solimões (approximately 40 km upstream of the mixing zone between the Rio Negro and the Rio Solimões, 03°21′19.7″ S, 060°12′40.1″ W), which was visited between 30 November and 5 December 2023. At both locations, the vessel was moored to a floating house. Experiments were conducted in an outdoor shaded area of the upper deck of the *Anna Clara*.

### 2.2 Experimental animals

Experiments were conducted with juvenile Tambaqui (*C. macropomum*), weighing 1.77 ± 0.05 g (*n* = 158), with a total length of 5.09 ± 0.9 cm (*n* = 48) and a fork length of 3.83 ± 0.07 cm (*n* = 48) (length was not measured for all experimental fish). They were obtained from the Santo Antonio farm, located in Rio Preto da Eva, near Manaus (AM, Brazil). This farm uses open systems that receive continuous water supply directly from the Amazon River basin, and fish are exposed to natural temperature and light cycles. The farm maintains a large amount of broodstock continuously enriched with wild‐caught adults from natural populations. Therefore, domestication effects should be relatively minimal, and the genetic variation in our study population is expected to be representative of wild populations.

Purchased fish were first transported to the Laboratory of Ecophysiology and Molecular Evolution (LEEM) of the Brazilian National Institute for Research of the Amazon (INPA), where they were kept for 2 weeks prior to being transferred to the research vessel where experiments were conducted. At INPA, the fish were held in 3000‐L polyethylene holding tanks supplied with aerated well water at ~28°C. The chemical composition of this INPA water [Ca = 11.9 ± 3.9 μM, Mg = 3.56 ± 0.79 μM, Na = 102 ± 12 μM, K = 25.2 ± 3.9 μM, Cl = 11 μM, DOC = 0.59 mg/L; mean ± standard error (SE) values] was similar to the ion‐poor water of the Rio Negro black water (Table 1) but with much lower levels of DOC. Once aboard the *Anna Clara*, fish were held in aerated water in 100‐L polyethylene tanks placed in a shaded area of the upper deck (outdoor). Half of the fish tank water was manually renewed every second day. Fish were fed every other day with commercial flakes (TetraMin, Tropical Flakes Fish Food, Spectrum Brands, Blacksburg, VA, USA) and were fasted for 24 h prior to experiments. Fish were maintained in INPA water until they were transported to field sites, where holding water was switched from INPA water to water collected freshly from the rivers at each of the field locations (compositions in Table 1) at least 2 days prior to experiments. This ≥2‐day period allowed fish to acclimate to the respective river ionic environments, so that they were at net Na^+^ and Cl^−^ balance prior to the start of experiments.

**TABLE 1:** Water quality summary in the two collected river waters.

Parameter	Rio Negro (‘black water’)	Rio Solimões (‘white water’)
T (°C) at collection date	31.0 ± 0.6 (*n* = 4)	31.3 ± 0.4 (*n* = 5)
pH	4.67 ± 0.12 (*n* = 9)	6.86 ± 0.14 (*n* = 5)
TSS (mg/L)	< 5 (*n* = 3)	56 ± 3 (*n* = 3)
Conductivity (μS/cm)	11.2 ± 0.5 (*n* = 8)	137 ± 3 (*n* = 4)
[Ca] (μM)	13.2 ± 2.4 (*n* = 4)	355 ± 15 (4)
[Mg] (μM)	8.07 ± 0.54 (*n* = 4)	86.8 ± 3.4 (4)
[Na] (μM)	45.8 ± 0.8 (*n* = 32)	352 ± 1 (*n* = 48)
[K] (μM)	23.4 ± 0.3 (*n* = 32)	36.9 ± 0.4 (*n* = 48)
[Cl (μM)	10.4 ± 1.3 (*n* = 32)	182 ± 3 (*n* = 48)
[DOC] (mg/L)	9.23 ± 0.09 (*n* = 12)	5.62 ± 0.27 (*n* = 7)

*Note*: Mean ± standard error (SE) (*n*).

Abbreviations: DOC, dissolved organic carbon; TSS, total dissolved solids.

All experimental procedures were approved by the Ethics Committee on Animal Experiments of INPA, under registration number 004/2018, in conformity with the Brazilian Ethics Committee on Animal Care (CONCEA) guidelines (RN 61/2023). The research was also authorized by the Brazilian Institute of Environment and Renewable Natural Resources (IBAMA) (SISBio number: 29837‐24).

### 2.3 Experimental waters

Experiments were conducted using unfiltered Rio Negro or Rio Solimões waters collected in large clean plastic carboys at least 200 m from the research vessel and other anthropogenic influences (compositions in Table 1, background Cu concentrations of 3–4 μg/L in Table 2). Experimental Cu solutions were prepared at least 24 h prior to use in experiments to allow full equilibration with ambient water chemistry. Copper was added to the river waters at 0 (control) and 200 μg/L in Rio Negro and 0, 200 and 600 μg/L in Rio Solimões from a 1000‐μg Cu/L stock solution prepared by dissolving CuSO_4_·5H_2_O (99% pure, Sigma Aldrich, St. Louis, Mo, USA) in ultrapure water (resistivity ≥18 Mohms cm). The 0 μg/L Cu treatment was the Cu control and corresponded to unspiked river water with about 3–4 μg/L of background total Cu concentration (Table 2). The 200 and 600 μg/L Cu concentrations represent very high contamination levels that may only occur in the vicinity of mining or industrial effluents in Amazonia (Braz‐Mota et al., 2024; Sampaio, 2000). These high concentrations were necessary to mount a fast physiological response in juvenile Tambaqui, which has been shown to be relatively tolerant to Cu at concentrations up to hundreds of μg/L dissolved Cu (Matsuo et al., 2005; Oliveira, 2003). Preliminary experiments during our trip showed that a 3‐h exposure to 200 μg/L of total Cu induced a Na^+^ imbalance in juvenile Tambaqui in Rio Negro but not in Rio Solimões, so we also added a higher treatment in this latter water. Also, note that in a preliminary experiment where 12 fish were exposed for 3 h to 200 μg/L of total Cu in 31.5°C Rio Negro water, then returned to Cu‐free Rio Negro water, all 12 fish were still alive a week later at the end of the research trip. This indicates that this acute Cu exposure was non‐lethal to the test organisms.

**TABLE 2:** Concentrations of total Cu, particulate, dissolved Cu, free Cu^2+^ and Cu‐DOC (in μg/L) in the treatments of each river water (control, 200 and 600 μg/L nominal Cu).

	Rio Negro (‘black water’)		Rio Solimões (‘white water’)		
	0 μg/L Cu	200 μg/L Cu	0 μg/L Cu	200 μg/L Cu	600 μg/L Cu
Total [Cu] (μg/L)	3.00 ± 0.18 (2)	200 ± 7 (2)	4.30 ± 0.95 (2)	190 ± 22 (2)	630 ± 150 (2)
Particulate [Cu] (μg/L)	0.05 ± 0.48	10 ± 12	2.2 ± 0.96	132 ± 22	455 ± 150
Dissolved [Cu] (μg/L)	2.95 ± 0.45 (6)	190 ± 10 (4)	2.10 ± 0.15 (2)	58 ± 13 (2)	175 ± 0.6 (2)
Free [Cu^2+^] (μg/L)	<0.1	15	<0.1	0.42	5.0
Cu‐DOC (μg/L)	<0.1	175	<0.1	57	168

*Note*: Total and dissolved Cu concentrations were measured in unfiltered and filtered (<0.45 μm) samples, respectively [mean ± standard error (SE) (*n*)]. Particulate Cu concentrations were obtained from the difference between measured total and dissolved Cu concentrations. Free Cu^2+^ and Cu‐DOC concentrations were modelled using WHAM (version VII). There was no 600 μg/L treatment in Rio Negro.

Abbreviations: Cu, copper; DOC, dissolved organic carbon.

### 2.4 Physiological fluxes and Cu gill bioaccumulation

For each Cu treatment (0, 200 or 600 μg/L), we had *n* = 8 replicates. Each replicate corresponded to a single fish individually exposed in a 150‐mL plastic cup initially filled with 100 mL of experimental water. Note that the low water volume to body mass ratio in the flux chambers was required for measurement sensitivity (Wood, 1992), and that confinement stress was expected to be minimal (dos Santos et al., 2021; Wood, 1992). Further, water ammonia levels over the flux period remained ≤100 μM, a safe level for this species (Wood et al., 2017). The fish chambers were randomly distributed in a water‐bath at either 31.5 ± 0.5°C (control temperature) or 35.5 ± 0.5°C (+4°C temperature treatment). The 31.5°C treatment corresponded to average river temperatures during the Nov–Dec 2023 trip. It can be considered as a representative water temperature maximum for the Amazon River basin, where typical water temperatures fluctuate between 28 and 31°C seasonally (Val & de Almeida‐Val, 1995). The 35.5°C treatment was representative of mean water temperatures reached in Lake Janauacá during the 2023 drought (Fleischmann et al., 2024). Water in the fish flux chambers was aerated with polyethylene capillary tubing and an air pump. Water temperature in the chambers was monitored throughout the experiment (Traceable 4378 thermometer, Cole‐Parmer, Vernon Hills, IL, USA) and kept within 0.5°C of the target temperature by occasionally adding heated water into the water‐bath.

Fish were transferred from their ~31°C acclimation tanks to their experimental containers (at 31.5 or 35.5°C) about 15 min prior to the start of the flux period. Then, 10 mL of water were collected at *t* = 0 h and 3 h for Na, Cl, K and total ammonia (NH_3_ + NH_4_^+^) analyses. At about 2.5 h of exposure, 20 μL of a radioactive ^22^Na stock solution (as NaCl in water, Eckert Ziegler, Valencia, CA, USA) were spiked in each container to obtain a final ^22^Na concentration of 0.002 μCi/mL, and 1 mL of water was collected after 10 min then again at 3 h of exposure, for Na and ^22^Na analyses to assess Na^+^ unidirectional fluxes in the shorter 2.5–3 h period (due to fast Na^+^ turnover rates). For ammonia analyses, 1‐mL aliquots of the 10‐mL samples were immediately kept in the freezer until analyses (< 72 h). Water samples were also collected at *t* = 0 and/or 3 h to measure pH and concentrations of DOC, Ca, Mg and total and dissolved Cu. For dissolved metal analyses, water was passed through a 0.45‐μm polyethersulfone membrane syringe filter (Fisher Scientific) and collected in a vial after pre‐conditioning the filter by discarding the first 10 mL of filtrate water. These samples were then acidified to 2% HNO_3_ (v/v) (trace metal grade, Fisher Scientific).

After the 3 h of exposure, water in each flux chamber was replaced with 20 mL of 1‐mM ethylenediaminetetraacetic acid (EDTA, ACS grade, Fisher Scientific) rinsing solution prepared in Cu‐free river water to desorb Cu loosely bound to the fish gill surface. After 5 min of rinsing, fish were euthanized with a solution of 250 mg/L of MS‐222 (Syndel Laboratories, Vancouver, BC, Canada) buffered to neutral pH with sodium bicarbonate. Immediately after death (within 2 min in MS‐222), fish were blotted dry and weighed (± 0.001 g). Their gills were dissected, weighed (± 0.001 g), then digested in 500 μL of 4 N HNO_3_ (trace metal grade, Fisher Scientific) in sealed 1.5‐mL plastic tubes on the boat deck under direct sunlight for about 3 days, with occasional vortexing.

### 2.5 Critical thermal maximum

Nine to ten fish were exposed together for 3 h to each Cu treatment (0, 200 or 600 μg/L) in 1.5 L of aerated experimental water at their control temperature 31 ± 0.5°C. After the 3‐h exposure, their CT_max_ was measured (*n* = 9–10 fish replicates). Note that because the aim of this specific experiment was to assess the effects of Cu on CT_max_, the Cu exposure was conducted only at the control temperature of 31°C.

To measure the CT_max_, fish were transferred to 2.1‐L plastic baskets submerged in an 80‐L plastic tank filled with aerated river water at river temperature. Each basket was divided into two individual compartments with a plastic separator, and one fish was placed per compartment. The baskets were closed with plastic nets to prevent escape. The fish were left to acclimate for 30 min, then the water temperature was gradually increased at a rate of 0.21 ± 0.02°C/min (*n* = 5 experiments) with a submersed heater (1000 W). Vigorous aeration and a circulation pump were used to prevent oxygen and thermal stratification during the thermal ramp. The temperature was monitored every 10 min during all trials using a temperature controller (Full Gage TIC‐17RGTi‐±0.1°C). The CT_max_ was identified as the temperature at which the fish exhibited a loss of equilibrium (LOE), defined here as the inability to maintain dorsal–ventral orientation for at least 10 s. After the experiment, each fish was measured (weight, total length and fork length).

### 2.6 Analytical techniques

Copper concentrations in collected water samples and in digested gill tissues were measured using inductively coupled plasma mass spectrometry (ICP‐MS Thermo ICap‐RQ). For this analysis, water samples were diluted to 0.2% (v/v) HNO_3_ (trace metal grade, Fisher) with ultrapure water. Concentrations of total aqueous Ca and Mg were measured using inductively coupled plasma atomic emission spectrometry (5110 dual‐view ICP‐AES, Agilent, Santa Clara, CA, USA). Concentrations of total aqueous K and Na were measured using flame atomic absorption spectrometry (Perkin Elmer AAnalyst 800 AA spectrophotometer, Norwalk, CT, USA). The ^22^Na in water samples was measured using liquid scintillation counting (Triathler LSC, Hidex, Mississauga, Canada). Each 1‐mL sample was added to 4 mL of scintillation cocktail (UltimaGold AB, PerkinElmer) and counted three times for 1 min using the instrument ^14^C counting window. Tests showed that quench was constant, so no correction was made. Aqueous total ammonia and Cl^−^ concentrations were measured with the colorimetric assays developed by Verdouw et al. (1978) and Zall et al. (1956), respectively, using an ultraviolet (UV)‐visible microplate spectrometer (SpectraMax M2, Molecular Devices Inc., Sunnyvale, CA, USA). Dissolved inorganic carbon (DIC) and DOC concentrations were measured using a total organic carbon analyser (Shimadzu TOC‐VCSH, Canby, OR, USA). Level of TSS in each water was measured by weighing (±0.1 mg) a 0.45‐μm cellulose ester membrane (Millipore, Sigma‐Aldrich) that had been dried at 80°C for 12 h, filtering 50 mL of water on it, then weighing it again after an additional identical drying step.

### 2.7 Data calculations

Net flux rates of Na^+^, Cl^−^, K^+^ and ammonia (in nmol/g/h) were calculated with Equation (1) (Wood, 1992):

(1) $$J_{\mathrm{net}}^{X}=\frac{\left({\left[X\right]}_{i}-{\left[X\right]}_{f}\right)\cdot V}{t\cdot m}$$

where [*x*]_*i*_ and [*x*]_*f*_ are the aqueous concentrations of the compound of interest (Na^+^, Cl^−^, K^+^ or ammonia) at the beginning and at the end of the flux period, respectively (in nmol/L); *V* is the flux volume (in L); *m* is the wet mass of the fish (in g); and *t* is the flux period duration (in h).

Unidirectional sodium influx and outflux rates (in nmol/g/h) were also calculated using Equations (2) and (3), respectively:

(2) JinNa=Na22i−Na22f·VSA·t·m

(3) $$J_{\text{out}}^{\mathrm{Na}}=J_{\mathrm{net}}^{\mathrm{Na}}-J_{\text{in}}^{\mathrm{Na}}$$

where [^22^Na]_*i*_ and [^22^Na]_*f*_ are the initial and final ^22^Na concentrations in the water (in CPM/L), respectively, and SA is the mean specific activity of ^22^Na in the water (in CPM/nmol of total Na).

In control (Cu‐free) treatments, we evaluated temperature sensitivity of physiological fluxes that were significantly different from zero, using temperature coefficients (Q_10_):

(4) $$Q_{10}={\left(\frac{J_{2}}{J_{1}}\right)}^{\left(\frac{10}{T_{2}-T_{1}}\right)}$$

where *J*_1_ and *J*_2_ are the net flux (Na^+^, Cl^−^, ammonia), influx or outflux (Na^+^ only) values at the two test temperatures *T*_1_ (35.1°C) and *T*_2_ (35.5°C), respectively.

Copper in the fish gills (Cu_gill_, in nmol/g wet weight) was calculated using Equation (5):

(5) $${\mathrm{Cu}}_{\text{gill}}=\frac{{\left[\mathrm{Cu}\right]}_{\text{digest}}\cdot V_{\text{digest}}}{m_{\text{gill}}}$$

where *V*_digest_ is the volume of the gill digestion (in L), *m*_gill_ is the wet mass of digested gills (in g) and [Cu]_digest_ is the Cu concentration in the gill digestion (in nmol/L).

Copper speciation in various experimental waters was modelled as detailed in Crémazy et al. (2016), where good agreement was found between measured Cu^2+^ concentrations (with a cupric ion‐selective electrode) and modelled Cu^2+^ concentrations in Amazon waters. This free ion species was of particular interest, as it is the best predictor of Cu bioavailability and toxicity (Duarte et al., 2024). Briefly, we used the thermodynamic calculation software WHAM (Windermere Humic Aqueous Model) (version VII) (Tipping et al., 2011), with measured water quality (Table 1) and Cu concentrations (Table 2) as input parameters. Alkalinity was nominal and taken from Crémazy et al. (2016). We assumed that DOM was 50% carbon in weight (Buffle, 1988), only 65% active in complexing Cu and composed of only fulvic acid (Bryan et al., 2002). Note that sensitivity tests indicated that changing to only humic acid‐type DOM had minimal influence on the Cu speciation predictions. The default log K_CuHCO3_ of 14.62 (Mattigod & Sposito, 1979) was replaced by 12.13 in the thermodynamic database, as recommended by the International Union of Pure and Applied Chemistry (Powell et al., 2007).

### 2.8 Statistics

Data are expressed as mean ± SE (*n*), where *n* = number of replicates. Data analysis was performed using GraphPad Prism (version 10.3.1), with a significance level of 0.05. Shapiro–Wilk tests confirmed the normal distribution of the data.

The CT_max_ values of control fish (0 μg/L Cu) in Rio Negro water versus Rio Solimões water were first compared with an unpaired *t*‐test. Then, effects of Cu exposure on CT_max_ were assessed separately in each water, using an unpaired *t*‐test in Rio Negro water (0 and 200 μg/L Cu) and a one‐way analysis of variance (ANOVA) for Rio Solimões water (0, 200, 600 μg/L Cu). For each physiological flux (*J* ^Na^_net_, *J* ^Na^_in_, *J* ^Na^_out_, *J* ^Cl^_net_, *J* ^K^_net_, *J* ^amm^_net_), and also Cu concentration in the gills, we evaluated the effects of Cu, temperature and their interaction in each water separately, using a two‐way ANOVA followed by a post hoc Fisher's least significant difference (LSD) test. In addition, for control fish (in Cu‐free water), net flux balance was evaluated at both temperatures, using one‐sample *t*‐test comparing the mean values to zero.

## 3 RESULTS

### 3.1 Water quality and copper speciation

Water quality in the two collected river waters is shown in Table 1. Rio Negro was 2.2 pH units more acidic, had lower concentrations of all major inorganic ions (12‐fold lower conductivity) and had a 1.6‐fold higher DOC concentration than Rio Solimões. The average water temperature at the two collection sites was similar in the two rivers (~31°C). Similar and relatively low background total Cu concentrations of 3–4 μg/L were measured in both river waters (Table 2). This baseline Cu was largely dissolved in Rio Negro and half‐associated with particles (>0.45 μm) in Rio Solimões (Table 2).

Total Cu concentrations measured in the two Cu‐treated waters (unfiltered samples) were within 95% of nominal test values of 200 and 600 μg/L (Table 2). Measured dissolved Cu concentration (<0.45‐μm filtered samples) represented 95% of total Cu in Rio Negro water and 32% of total Cu in Rio Solimões (Table 2). WHAM predicted that about 8.1% of dissolved Cu in the Rio Negro was in free ionic form (Cu^2+^) in the 200 μg/L treatment (Table 2). In Rio Solimões, this Cu^2+^ proportion was only 0.7% and 2.9% in the 200 and 600 μg/L treatments, respectively (Table 2). In both Cu‐spiked river waters, Cu bound to DOC was the main dissolved metal form (between 91% and 99% of dissolved Cu). Overall, because of the larger binding of Cu to particles and DOC in Rio Solimões than in Rio Negro, even the highest Cu treatment of 600 μg/L in Rio Solimões had a 67% lower Cu^2+^ concentration (5 μg/L) than in Rio Negro (15 μg/L) (Table 2).

### 3.2 Copper accumulation in fish gills

**FIGURE 1:**
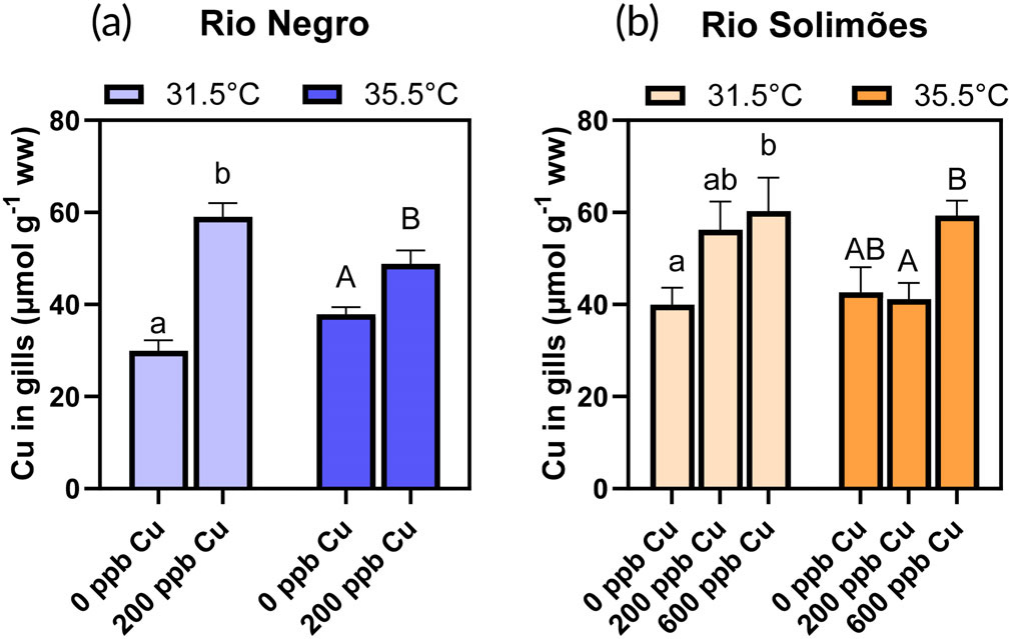
Copper (Cu) in gills of *Colossoma macropomum* after a 3‐h exposure to either 0, 200 or 600 μg/L of total Cu at 31.5 or 35.5°C in (a) Rio Negro water and (b) Rio Solimões water. For a given temperature, bars sharing a common letter are not significantly different (Cu effect) [Fisher's least significant difference (LSD) test]. Lower case letters are used for comparisons at 31.5°C and upper case letters for comparisons at 35.5°C. For a given Cu concentration, asterisks on a 35.5°C bar indicate a difference with the 31.5°C bar (temperature effect) (Fisher's LSD test). Data presented as mean ± standard error (SE), *n* = 7–8 fish.

Temperature (31.5 vs. 35.5°C) did not affect the Cu concentration in gills of Tambaqui (Figure 1) exposed in either Rio Negro (*F*_1,27_ = 0.20, *p* = 0.66) or Rio Solimões (*F*_1,40_ = 1.20, *p* = 0.28) (two‐way ANOVAs). We measured background Cu concentrations in control fish gills of 34 ± 2 (*n* = 15) μmol/g ww in Rio Negro (Figure 1a) and 42 ± 3 (*n* = 15) μmol/g ww in Rio Solimões (Figure 1b). A 3‐h Cu exposure led to significant branchial Cu accumulation in Rio Negro (*F*_1,27_ = 61.2, *p* < 0.0001) and in Rio Solimões (*F*_2,40_ = 6.90, *p* = 0.0027) (two‐way ANOVAs) (Figure 1). More precisely, in Rio Negro (Figure 1a), a 3‐h exposure to 200 μg/L of total Cu led to a 1.60‐fold increase in Cu gill concentration. In Rio Solimões (Figure 1b), a 3‐h exposure to 600 μg/L of total Cu led to a 1.45‐fold increase in Cu gill concentration.

### 3.3 Fish physiological fluxes

**FIGURE 2:**
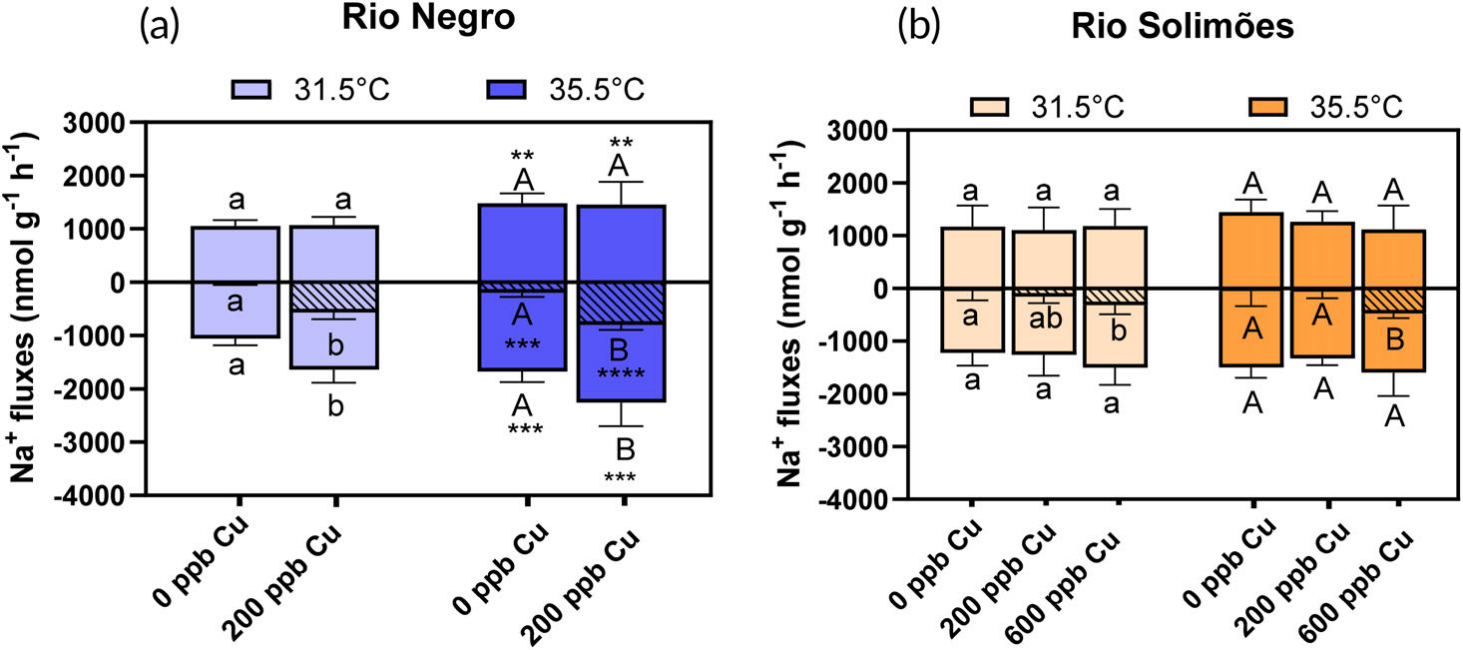
Sodium fluxes in *Colossoma macropomum* in (a) Rio Negro water and (b) Rio Solimões water over a 3‐h exposure to either 0, 200 or 600 μg/L of total Cu at 31.5 or 35.5°C. Plain bars above and below the *y*‐axis represent influx and outflux, respectively, and hatched bars represent the net flux. For a given temperature and flux type, bars sharing a common letter are not significantly different [copper (Cu) effect] [Fisher's least significant difference (LSD) test]. For a given Cu concentration and flux type, asterisks on a 35.5°C bar indicate a difference with the 31.5°C bar (temperature effect) (Fisher's LSD test, with **p < 0.01, ***p < 0.001, ****p < 0.0001). Lower case letters are used for comparisons at 31.5°C and upper case letters for comparisons at 35.5°C. Data presented as mean ± standard error (SE), *n* = 8 fish.

**FIGURE 3:**
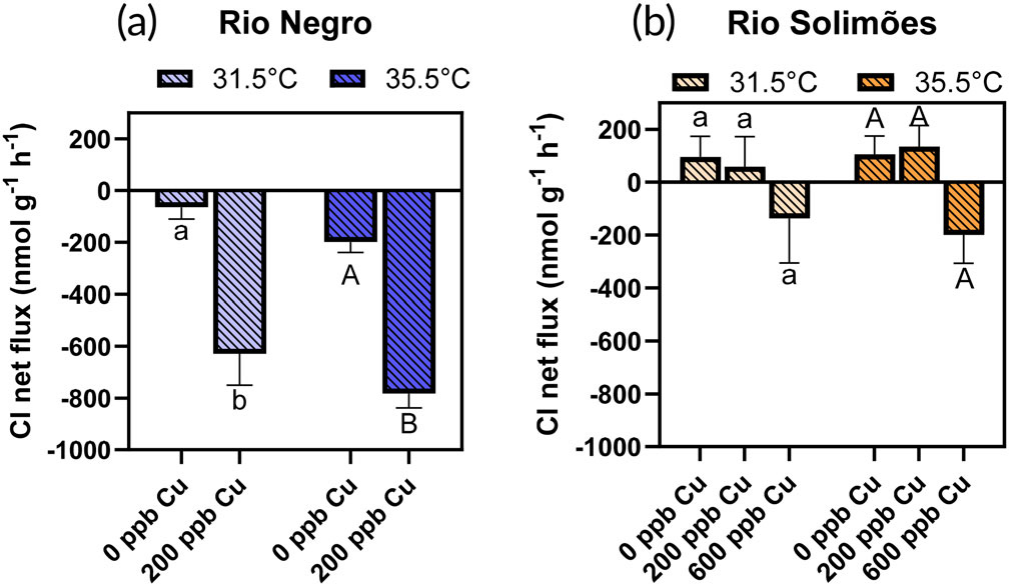
Chloride net fluxes in *Colossoma macropomum* in (a) Rio Negro water and (b) Rio Solimões water, over a 3‐h exposure to either 0, 200 or 600 μg/L of total Cu at 31.5 or 35.5°C. For a given temperature, bars sharing a common letter are not significantly different [copper (Cu) effect] [Fisher's least significant difference (LSD) test]. Lower case letters are used for comparisons at 31.5°C and upper case letters for comparisons at 35.5°C. For a given Cu concentration, asterisks on a 35.5°C bar indicate a difference with the 31.5°C bar (temperature effect) (Fisher's LSD test). Data presented as mean ± standard error (SE), *n* = 8 fish.

**FIGURE 4:**
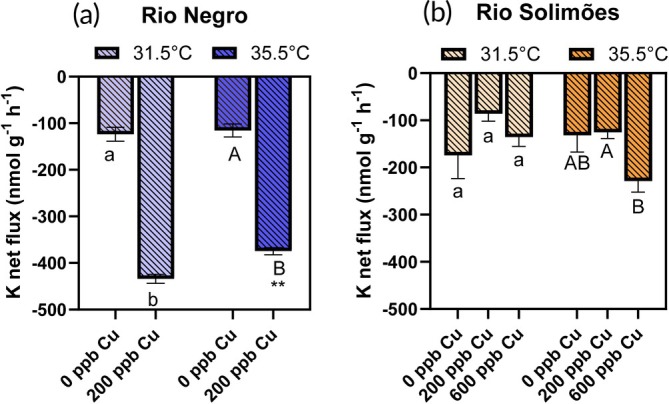
Potassium net fluxes in *Colossoma macropomum* in (a) Rio Negro water and (b) Rio Solimões water, over a 3‐h exposure to either 0, 200 or 600 μg/L of total copper (Cu) at 31.5 or 35.5°C. For a given temperature, bars sharing a common letter are not significantly different (Cu effect) (Fisher's least significant difference (LSD) test]. Lower case letters are used for comparisons at 31.5°C and upper case letters for comparisons at 35.5°C. For a given Cu concentration, asterisks on a 35.5°C bar indicate a difference with the 31.5°C bar (temperature effect) (Fisher's LSD test, with **p < 0.01). Data presented as mean ± SE, *n* = 8 fish.

**FIGURE 5:**
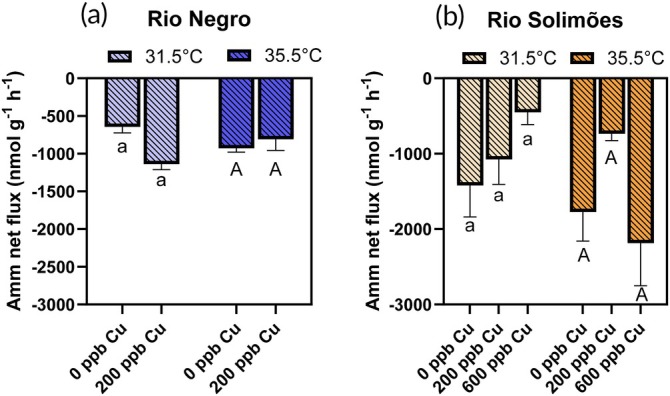
Ammonia net fluxes in *Colossoma macropomum* in (a) Rio Negro water and (b) Rio Solimões water, over a 3‐h exposure to either 0, 200 or 600 μg/L of total copper (Cu) at 31.5 or 35.5°C. For a given temperature, bars sharing a common letter are not significantly different (Cu effect) [Fisher's least significant difference (LSD) test]. Lower case letters are used for comparisons at 31.5°C and upper case letters for comparisons at 35.5°C. Data presented as mean ± standard error (SE), *n* = 8 fish.

Physiological fluxes of Na^+^, Cl^−^, K^+^ and ammonia are presented in the two river waters in Figures 2, 3, 4, 5, respectively. No fish died during these flux experiments, which were also used to measure Cu‐gill concentrations.

#### 3.3.1 Sodium unidirectional flux rates

For fish exposed in Rio Negro water (Figure 2a), we observed strong temperature effects on *J* ^Na^_net_ (*F*_1,28_ = 43.3, *p* < 0.0001), *J* ^Na^_in_ (*F*_1,28_ = 20.7, *p* < 0.0001) and *J* ^Na^_out_ (*F*_1,28_ = 38.7, *p* < 0.0001) (two‐way ANOVAs), with absolute values increasing from 31.5 to 35.5°C in both control and Cu‐exposed fish (*p* < 0.01, Fisher's LSD). Control fish at 31.5°C were at Na^+^ balance, as evidenced by a *J* ^Na^_net_ of zero (*p* = 0.95, one‐sample *t*‐test) resulting from equal Na^+^ influx and outflux rates. However, control fish at 35.5°C showed significant net Na^+^ loss over the 3‐h flux period (*p* = 0.0002, one‐sample t‐test), as temperature sensitivity was higher for *J* ^Na^_out_ (Q_10_ = 3.16) than for *J* ^Na^_in_ (Q_10_ = 2.32) (Table 3). Copper exposure also had a strong effect on *J* ^Na^_net_ (*F*_1,28_ = 318, *p* < 0.0001) and *J* ^Na^_out_ (*F*_1,28_ = 34.2, *p* < 0.0001) (two‐way ANOVAs), with values becoming more negative from 0 to 200 μg/L Cu at both temperatures (*p* < 0.001, Fisher's LSD). On the contrary, Cu did not affect *J* ^Na^_in_ (*F*_1,28_ = 6e‐4, *p* = 0.98, two‐way ANOVA). There was no interaction between temperature and copper for any of the Na^+^ flux rates, that is, for *J* ^Na^_net_ (*F*_1,28_ = 0.32, *p* = 0.58), *J* ^Na^_in_ (*F*_1,28_ = 0.04, *p* = 0.84) and *J* ^Na^_out_ (*F*_1,28_ = 3e‐5, *p* = 0.99) (two‐way ANOVAs).

**TABLE 3:** Q_10_ values for physiological flux rates of *Colossoma macropomum* measured in Rio Negro or Rio Solimões waters.

	Rio Negro (‘black water’)	Rio Solimões (‘white water’)
Q_10_ of *J* ^Na^in	2.32	1.69
Q_10_ of *J* ^Na^out	3.16	1.66
Q_10_ of *J* ^Na^net	–	–
Q_10_ of *J* ^Cl^net	–	–
Q_10_ of *J* ^K^net	0.85	0.49
Q_10_ of *J* ^amm^net	2.52	1.74

*Note*: These Q_10_ values were calculated using Equation (4) from the mean control data (Cu‐free water) between 31.5 and 35.5°C in Figures 2, 3, 4, 5. Q_10_ values for *J* ^Na^net and *J* ^Cl^net could not be calculated because of a zero *J*_net_ value.

In Rio Solimões (Figure 2b), contrary to Rio Negro, no temperature effect was observed on Na^+^ flux rates, that is, for *J* ^Na^_net_ (*F*_1,41_ = 0.16, *p* = 0.69), *J* ^Na^_in_ (*F*_1,41_ = 1.47, *p* = 0.23) and *J* ^Na^_out_ (*F*_1,41_ = 2.59, *p* = 0.12) (two‐way ANOVAs). Control fish had Q_10_ values of 1.69 and 1.66 for *J* ^Na^_in_ and *J* ^Na^_out_, respectively (Table 3), and they maintained their Na^+^ balance at both temperatures (*p* = 0.43 at 31.5°C and *p* = 0.62 at 35.5°C, one‐sample *t*‐tests). Copper effects in Rio Solimões were not as marked as in Rio Negro water. Indeed, Cu only affected *J* ^Na^_net_ (*F*_2,41_ = 18.3, *p* < 0.0001, two‐way ANOVA), with net Na^+^ loss rates increasing in fish exposed to 600 μg/L (vs. unexposed fish) at both temperatures (*p* < 0.01, Fisher's LSD).

#### 3.3.2 Chloride net flux rates

The two‐way ANOVA did not detect a significant temperature effect on chloride net fluxes of Rio Negro fish (*F*_1,28_ = 3.89, *p* = 0.058), despite an apparent trend towards more negative *J* ^Cl^_net_ as temperature increased (Figure 3a). In fact, one‐sample *t*‐tests found that although control fish were at Cl^−^ balance at 31.5°C (*p* = 0.19), there was a significant net loss rate at 35.5°C (*p* = 0.0015) similar to the Na^+^ fluxes. A strong Cu effect was observed (*F*_1,28_ = 61.4, *p* < 0.0001, two‐way ANOVA), with Cu addition promoting a 4‐fold to 10‐fold increase in Cl^−^ net loss rates at 31.5 and 35.5°C, respectively (*p* < 0.0001, Fisher's LSD).

In Rio Solimões (Figure 3b), the absence of temperature effect on *J* ^Cl^_net_ was clear (*F*_1,41_ = 7e‐3, *p* = 0.93, two‐way ANOVA), with Cl^−^ balance being observed in control fish at both temperatures (*p* = 0.26 at 31.5°C and *p* = 0.18 at 35.5°C, one‐sample *t*‐tests). As in the Rio Negro water, a Cu effect was also observed, but it was relatively weak (*F*_2,41_ = 4.10, *p* = 0.0239, two‐way ANOVA) and not apparent in the pair‐wise comparisons (*p* > 0.05, Fisher's LSD).

#### 3.3.3 Potassium net flux rates

Net loss of K^+^ was evident in control fish (no Cu) exposed in both waters at both temperatures (Figure 4) (*p* < 0.01, one‐sample *t*‐tests). In Rio Negro water (Figure 4a), the two‐way ANOVA found a temperature effect (*F*_1,28_ = 8.00, *p* = 0.0085) and a Cu effect (*F*_1,28_ = 567, *p* < 0.0001), with a significant interaction between the two variables (*F*_1,28_ = 4.67, *p* = 0.0395). There was a 3.4‐fold promotion of K^+^ net loss rate in the Cu‐spiked water at both temperatures (*p* < 0.0001, Fisher's LSD). Further, the K^+^ net loss rate in Cu‐exposed fish was about 16% lower at 35.5°C than at 31.5°C (*p* = 0.0015, Fisher's LSD). In Rio Solimões water (Figure 4b), the temperature effect was no longer observed (*F*_1,41_ = 1.56, *p* = 0.22, two‐way ANOVA), and the Cu effect was much weaker (*F*_2,41_ = 3.37, *p* = 0.044, two‐way ANOVA) than in Rio Negro water. At 35.5°C, K^+^ net loss was larger at 600 μg/L of Cu than at 200 μg/L of Cu (*p* = 0.042, Fisher's LSD).

#### 3.3.4 Ammonia net flux rates

Ammonia excretion was measured over the 3‐h flux period in control fish (no Cu) exposed in both waters at both temperatures (Figure 5) (*p* < 0.05, one‐sample *t*‐tests). In Rio Negro, *J* ^amm^_net_ was not affected by temperature (*F*_1,28_ = 0.05, *p* = 0.82) or Cu (*F*_1,28_ = 3.98, *p* = 0.056) (two‐way ANOVAs) (Figure 5b). Likewise in Rio Solimões (Figure 5b), *J* ^amm^_net_ was not affected by temperature (*F*_1,41_ = 3.84, *p* = 0.057) or Cu (*F*_2,41_ = 1.79, *p* = 0.18) (two‐way ANOVAs). Despite the absence of detectable temperature effects, we calculated Q_10_ values of 2.52 and 1.74 on *J* ^amm^_net_ in control fish in Rio Negro and Rio Solimões, respectively (Table 3).

### 3.4 Fish upper thermal tolerance

**FIGURE 6:**
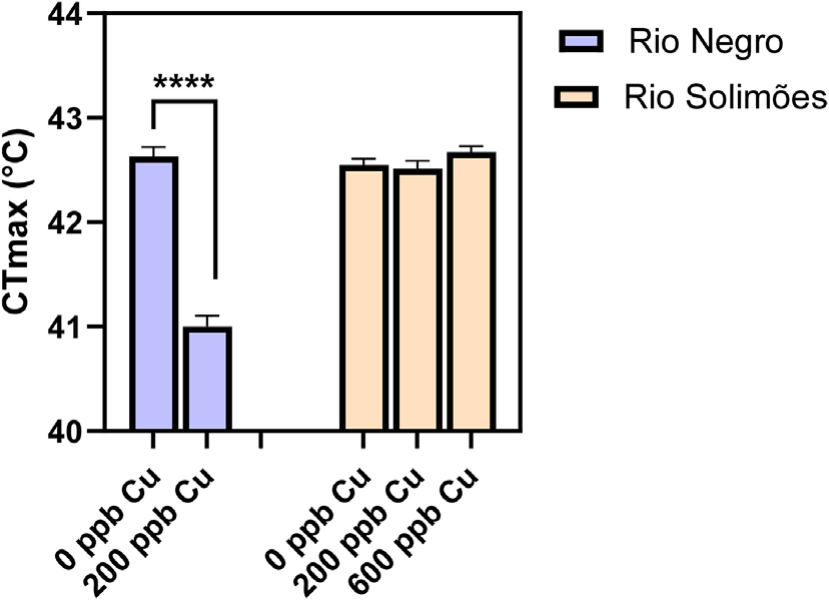
Critical thermal maximum after a 3‐h exposure of Tambaqui (*Colossoma macropomum*) to 0, 200 or 600 μg/L of total copper (Cu) in Rio Negro water (in blue) or Rio Solimões water (in beige). The asterisks show a significant difference (****p < 0.0001, unpaired *t*‐test). Data presented as mean ± standard error (SE) (*n* = 9–10 fish).

The CT_max_ of juvenile Tambaqui (Figure 6) measured in Cu‐free (control) water was 42.63 ± 0.09°C (*n* = 9) in Rio Negro and 42.52 ± 0.06°C (*n* = 10) in Rio Solimões, with no significant difference between the two waters (*p* = 0.4, unpaired *t*‐test). No fish died from the Cu exposure leading up to the CT_max_ measurement. In Rio Negro, a 3‐h exposure to 200 μg/L of total Cu led to a significant CT_max_ reduction by 1.63°C down to 41.00 ± 0.10°C (*n* = 9) (*p* < 0.0001, unpaired *t*‐test) (Figure 6). On the contrary, in Rio Solimões, a 3‐h exposure to up to 600 μg/L of total Cu had no effect on CT_max_ (*F*_2,27_ = 1.62, *p* = 0.2, one‐way ANOVA) (Figure 6).

## 4 DISCUSSION

The goal of this study was to investigate the effects, both direct and interactive, of elevated Cu concentration and temperature on juvenile Tambaqui exposed in black and white river waters. We found that Cu disrupted ion regulation and decreased upper thermal tolerance in fish exposed in Rio Negro water, but not in Rio Solimões water where Cu bioavailability was lower. Additionally, a +4°C rise in water temperature accelerated both Na^+^ influx and efflux rates in fish in Rio Negro water, but not in Rio Solimões water. The worst‐case scenario for Na^+^ regulation was observed under joint Cu and heat exposures. Altogether, this study suggests a cumulative physiological interaction between Cu and heat stress, which is of ecological relevance for fish health in the Amazon.

### 4.1 Copper effects on Tambaqui

#### 4.1.1 Differences between the two waters

The gill bioaccumulation (Figure 1) and effects of Cu (Figures 2, 3, 4, 5, 6) were generally greater in fish exposed in Rio Negro water than in Rio Solimões water even with the threefold higher total Cu concentration in Rio Solimões (600 vs. 200 μg/L of total Cu). This could be explained by Cu bioavailability differences in the two waters (Table 2) due to the distinct water quality differences in the two rivers (Table 1). Indeed, it is well known that Cu bioavailability and toxicity to aquatic organisms are best predicted by the aqueous concentration of the free Cu^2+^ ion rather than by the total Cu concentration (Campbell, 1995; Duarte et al., 2024). In agreement, we estimated that Cu^2+^ concentration was three times higher in Rio Negro than in Rio Solimões. This was due to the larger fraction of total Cu that was bound to be the abundant suspended solids in the Rio Solimões (68% vs. 5% of total Cu) and to the larger Cu‐DOC binding in the Rio Solimões (96% vs. 92% of total dissolved Cu) compared to the Rio Negro. This Cu‐DOC complexation difference might be counter‐intuitive, as DOC concentration was 1.6‐fold greater in Rio Negro than in Rio Solimões (Table 1). However, the 156‐fold higher acidity of the Rio Negro (>2 pH units lower than in the Rio Solimões) limited Cu binding to DOC in favour of Cu^2+^ formation. The present results contrast with our previous Cu studies with cardinal tetra (Crémazy et al., 2016, 2019), where 96‐h LC_50_ (total Cu concentration leading to 50% lethality) was twofold higher in unfiltered Rio Negro than in unfiltered Rio Solimões. However, this discrepancy can be explained by the different compositions of the test waters used in these previous studies (Rio Negro water with a higher pH of 5.6 and Rio Solimões water with a lower DOC of 2.8 mg/L, collected in November 2015, likely due to different hydrological and climate conditions (Martineac et al., 2024).

#### 4.1.2 Copper effects on physiological fluxes

Firstly, fish in the control (no Cu added) in both the 31.5°C Rio Negro and Rio Solimões waters were at Na^+^ balance (Figure 2) and Cl^−^ balance (Figure 3) during the experiments. This indicates that the 2‐day river water acclimation period, while relatively short due to fieldwork constraints, was sufficient time to allow the fish to regulate their ion transport systems to their new ionic environment.

Acute Cu exposure disrupted Na^+^ homeostasis in juvenile Tambaqui. This result was anticipated, as Na^+^ homeostasis is considered the key acute toxicity target of Cu in freshwater organisms, with death occurring when the blood plasma loses about 30% of its exchangeable Na^+^ pool (Grosell, 2011). Death did not occur during our 3‐h exposures because total Na^+^ losses (Figure 2a) were only about 5% of the estimated exchangeable Na^+^ pool (see table 1 of Wood, 1992). This Na^+^ net loss occurred via increase in Na^+^ branchial efflux rate (*J* ^Na^_out_) and not via inhibition of Na^+^ branchial uptake rate (*J* ^Na^_in_) (Figure 2a). This conforms with a previous study on acute Cu effects to juvenile Tambaqui (Matsuo et al., 2005). More generally, this agrees with findings that Characiformes have a highly specialized branchial Na^+^ uptake system with low sensitivity to acidity and Cu (Braz‐Mota et al., 2018; Crémazy et al., 2022; Gonzalez et al., 2002). We also found that Cu stimulated Cl^−^ (Figure 3a) and K^+^ (Figure 4a) net loss rates, which are other commonly reported secondary mechanisms of acute Cu toxicity (Grosell, 2011). Finally, Cu did not impair ammonia excretion in juvenile Tambaqui (Figure 5a), in accordance with previous studies on Amazon fish (Crémazy et al., 2016; Wood et al., 2014).

#### 4.1.3 Copper effects on acute upper thermal tolerance

The Tambaqui is a eurythermal fish that has a higher upper thermal tolerance than most Amazonian fishes tested to date (Campos et al., 2018, 2019; Jung et al., 2020; Kochhann et al., 2021). Our CT_max_ value in Cu‐free water was 42.6°C (Figure 6), which is very similar to previously reported values (42–42.9°C) on juvenile Tambaqui acclimated to similar water temperatures (Jung et al., 2020; Lapointe et al., 2018). Therefore, we did not expect acute thermal stress in fish exposed to 35.5°C for only 3 h in our study. Nevertheless, this latter temperature is 4°C above the typical water temperature maximum of ~31°C in the Amazon River basin (Val & de Almeida‐Val, 1995), and prolonged exposures above the latter value have been shown to be detrimental to Tambaqui (Amanajás & Val, 2023; Lapointe et al., 2018). Notably, Lapointe et al. (2018) showed that juvenile Tambaqui exposed at 35°C for 21 days had reduced growth and survival compared to fish exposed at 31°C. In fact, Tambaqui optimal temperature appears to be around 27°C (Amanajás & Val, 2023; Merola & Pagán‐Font, 1988).

In the present study, Tambaqui that had been acutely exposed to Cu in Rio Negro (where Cu bioavailability was high) had a reduced upper thermal tolerance, as evidenced by a 1.6°C decrease in CT_max_ (Figure 6). This might be due to effects of Cu on energy metabolism and the relationship between temperature tolerance and aerobic metabolism. Indeed, according to the concept of oxygen‐ and capacity‐limited thermal tolerance (OCLTT) (Pörtner, 2001), upper thermal limits occur when there is a mismatch between oxygen supply and oxygen demand in aquatic ectotherms. However, Cu (and other metals) has been shown to increase oxygen demand (e.g., via increased cellular metabolism related to cellular detoxification and repair) and to reduce oxygen supply (e.g., via damage to respiratory epithelia or decreased heart rate) in aquatic organisms (as reviewed by Sokolova & Lannig, 2008). The resulting reduction in aerobic scope would be expected to reduce thermal tolerance, according to the OCLTT theory. Among the few studies that have investigated the effect of metals on the CT_max_ of fish, the majority of them have indeed reported a decrease in CT_max_ following metal exposure (Dale Becker & Wolford, 1980; Dornelles Zebral et al., 2019; Lydy & Wissing, 1988; Paladino & Spotila, 1978), but two have found an opposite effect with Cu and fish (Kumar et al., 2023; Mottola et al., 2022). Notably, Mottola et al. (2022) found that the CT_max_ of three‐spined sticklebacks was increased by ~1.5°C after a 1‐week copper exposure (100 μg/L) in a sex‐specific manner. The authors suggested that this enhanced upper tolerance may result from a cross‐protection interaction (Rodgers & Gomez Isaza, 2021), or from an enhanced oxygen delivery to tissues from known positive effects of Cu on haemoglobin and haematocrit levels (Dethloff et al., 1999), and/or on the expression of hypoxia‐inducible factor 1‐alpha (HIF‐1alpha) (which enhances capillary density) (Nikinmaa & Rees, 2005).

### 4.2 Temperature effects on Tambaqui

#### 4.2.1 Temperature effects on physiological fluxes

In Cu‐free water, raising temperature from 31.5 to 35.5°C increased Na^+^ unidirectional fluxes in juvenile Tambaqui, but this effect was only significant in Rio Negro water (Figure 2a). In this water, Q_10_ values for *J* ^Na^_in_ and *J* ^Na^_out_ were 3.16 and 2.32, respectively (Table 3). To our knowledge, only four other studies have reported on the effects of temperature on *J* ^Na^_in_ and *J* ^Na^_out_ in freshwater fish, with similar mean Q_10_ values between 1.7 and 3.6 (Gonzalez & McDonald, 2000; Macpherson & Crémazy, 2024; Maetz, 1972; Wood et al., 2026). Notably, Wood et al. (2026) found a similar temperature dependence on *J* ^Na^_in_ and *J* ^Na^_out_ in juvenile Tambaqui exposed in INPA water, with Q_10_ values around 2.0 (range: 1.5–2.7). In our study, the lower Q_10_ value of 1.7 observed in Rio Solimões water for these two fluxes (Table 3) remains within the literature range and does suggest a temperature dependency, but the two‐way ANOVA did not detect a temperature effect (Figure 2b). We cannot explain why the temperature dependency of *J* ^Na^_in_ and *J* ^Na^_out_ appears lower in fish in Rio Solimões water compared to fish in Rio Negro water. Indeed, the Na^+^ unidirectional fluxes were very similar in both Cu‐free river waters, and similar rates were also measured in INPA water by Wood et al. (2026). These comparable rates suggest that a similar amount of energy is expended towards Na^+^ homeostasis in the different waters despite their distinct compositions. Interestingly, *J* ^Na^_net_ became slightly negative in Rio Negro water at 35.5°C, as *J* ^Na^_out_ was more sensitive to warming than *J* ^Na^_in_. On the contrary, temperature did not affect *J* ^Na^_net_ in fish in Rio Solimões (this study) and in INPA water (Wood et al., 2026). This latter absence of temperature effect better agrees with studies on other species (Evans, 1969; Macpherson & Crémazy, 2024; Motais & Isaia, 1972; Onukwufor & Wood, 2020) and with the branchial regulation of the two other ions, Cl^−^ (Figure 3) and K^+^ (Figure 4), measured in this study. Clearly, there is a need for additional studies to understand how temperature affects Na^+^ homeostasis in freshwater fish under various exposure conditions.

In our study, we did not detect a significant temperature effect on ammonia excretion (Figure 5), even though Q_10_ values suggest some temperature sensitivity (1.74–2.52) (Table 3). This agrees with the data of Wood et al. (2017), who reported that the Tambaqui has a well‐regulated ammonia metabolism (Q_10_ ~ 1.5) between 28 and 33°C (as opposed to most teleosts; Wood, 2001), but that this regulation breaks down (Q_10_ ~ 3.8) between 33 and 38°C.

#### 4.2.2 Temperature effects on Cu bioaccumulation and effects

Temperature had no significant effects on acute Cu gill accumulation under the conditions tested in our study (Figure 1). Increases in temperature are expected to increase biochemical reaction rates, according to the Arrhenius law (Schulte et al., 2011). As bioaccumulation is the net result of uptake and elimination processes, the absence of temperature effect may indicate a similar thermal dependency on the rates of metal uptake and elimination in our study. Additionally, at least in part, this may be a resolution of detection issue, as background Cu concentrations in gill tissue under control conditions were substantial (Figure 1), reflecting the essentiality of this metal in normal cellular functions (Grosell, 2011). Similar observations have been made in past temperature/metal interaction studies with aquatic ectotherms (Macpherson & Crémazy, 2024; Mattsson & Crémazy, 2023), although the majority of studies report an increase in metal bioaccumulation with elevated temperatures (Sokolova & Lannig, 2008).

Likewise, temperature had limited effects on Cu physiological effects in juvenile Tambaqui, so we could not elucidate specific mechanisms of interaction between these two stressors. In Rio Negro, these two stressors separately affected ion regulation. Indeed, warming increased the rates of baseline Na^+^ fluxes in fish, whereas Cu increased *J* ^Na^_out_ by a similar factor of ~1.4‐fold at 31.5 and at 35.5°C (Figure 2a). In our study, the absence of thermal effect on Cu physiological effects is in good agreement with the absence of thermal effect on Cu gill accumulation data (Figure 1a). However, a biologically meaningful interaction was observed as Cu and heat acted jointly to yield a worst‐case scenario on Na^+^ regulation. Indeed, *J* ^Na^_out_ and *J* ^Na^_net_ were at their most negative in Cu‐exposed fish at 35.5°C. In agreement with this observation, the review of Sokolova and Lannig (2008) indicated that an increase in temperature increased metal toxicity in 80% of the studies and led to no change in 15% of the studies (*n* = 115).

### 4.3 Future directions

The present study provides evidence that Cu worsens temperature effects on juvenile Tambaqui, with a decrease in CT_max_ following Cu exposure. Further, in Rio Negro where Cu effects were more evident, both elevating water temperature and Cu concentration appear to jointly diminish the ability of an Amazon fish to regulate their ion levels. Further studies are needed to confirm this latter observation, as temperature effects on Na^+^ balance were only observed in Rio Negro.

Overall, more studies are needed to assess how metal pollution affects the capacity of Amazon fish to respond to an acute temperature challenge associated with a heatwave or to a slower increase in ambient temperature associated with global warming. Conversely, more studies are needed to assess how acute and chronic warming may affect the sensitivity of Amazon fish to chemical pollutants. We recommend that future studies examine chronic metal exposure, using lower metal concentration that are more likely to occur in the Amazon. Further, we recommend studying fish species that are more sensitive to both metals and heat and, thus, are at greater risk of the dual threat of pollution and global warming (e.g., *Carnegiella strigata*, A. Günther 1864) (Braz‐Mota & Val, 2024; Duarte et al., 2024).

## AUTHOR CONTRIBUTIONS

Anne Crémazy: conceptualization, resources, formal analysis, investigation, methodology, validation, visualization, writing – original draft, writing – review and editing. Carolyn Morris and Susana Braz‐Mota: conceptualization, investigation, methodology, writing – review and editing. Chris M. Wood: conceptualization, methodology, resources, writing – review and editing. Rafael M. Duarte: investigation, methodology, writing – review and editing. Ora E. Johannsson: investigation, writing – review and editing. Adalberto L. Val: project administration, funding acquisition, resources, writing – review and editing.

## FUNDING INFORMATION

This study was funded by Natural Sciences and Engineering Research Council of Canada (NSERC) Discovery Grants awarded to Anne Crémazy (RGPIN‐2019‐04400) and to Chris M. Wood (RGPIN‐2023‐03714). This study was partially funded by CNPq (Brazilian National Research Council), CAPES (Coordination of Superior Level Staff Improvement) and FAPEAM (Amazonas State Research Foundation) via funding for INCT ADAPTA (CNPQ process no. 465540/2014‐7, CAPES – finance code 001 and FAPEAM process 062.01187/2017) to Adalberto L. Val. Adalberto L. Val is the recipient of a research fellowship from CNPq.
